# Therapeutic hypothermia attenuates physiologic, histologic, and metabolomic markers of injury in a porcine model of acute respiratory distress syndrome

**DOI:** 10.14814/phy2.15286

**Published:** 2022-05-04

**Authors:** Sarah A. Angus, William R. Henderson, Mohammad M. Banoei, Yannick Molgat‐Seon, Carli M. Peters, Hanna R. Parmar, Donald E. G. Griesdale, Mypinder Sekhon, Andrew William Sheel, Brent W. Winston, Paolo B. Dominelli

**Affiliations:** ^1^ 8430 Department of Kinesiology University of Waterloo Waterloo Ontario Canada; ^2^ 8166 Division of Critical Care Medicine Department of Medicine Faculty of Medicine University of British Columbia Vancouver British Columbia Canada; ^3^ 2129 Department of Critical Care Medicine University of Calgary Calgary Alberta Canada; ^4^ Department Kinesiology and Applied Health University of Winnipeg Winnipeg Manitoba Canada; ^5^ 8166 School of Kinesiology University of British Columbia Vancouver British Columbia Canada; ^6^ 8166 Department of Anesthesiology Pharmacology & Therapeutics University of British Columbia Vancouver British Columbia Canada; ^7^ 2129 Department of Critical Care Medicine University of Calgary Calgary Alberta Canada; ^8^ 2129 Departments of Medicine and Biochemistry & Molecular Biology University of Calgary Calgary Alberta Canada

**Keywords:** hemodynamics, inflammation, lung injury, respiratory mechanics

## Abstract

Acute respiratory distress syndrome (ARDS) is a lung injury characterized by noncardiogenic pulmonary edema and hypoxic respiratory failure. The purpose of this study was to investigate the effects of therapeutic hypothermia on short‐term experimental ARDS. Twenty adult female Yorkshire pigs were divided into four groups (*n* = 5 each): normothermic control (C), normothermic injured (I), hypothermic control (HC), and hypothermic injured (HI). Acute respiratory distress syndrome was induced experimentally via intrapulmonary injection of oleic acid. Target core temperature was achieved in the HI group within 1 h of injury induction. Cardiorespiratory, histologic, cytokine, and metabolomic data were collected on all animals prior to and following injury/sham. All data were collected for approximately 12 h from the beginning of the study until euthanasia. Therapeutic hypothermia reduced injury in the HI compared to the I group (histological injury score = 0.51 ± 0.18 vs. 0.76 ± 0.06; *p *= 0.02) with no change in gas exchange. All groups expressed distinct phenotypes, with a reduction in pro‐inflammatory metabolites, an increase in anti‐inflammatory metabolites, and a reduction in inflammatory cytokines observed in the HI group compared to the I group. Changes to respiratory system mechanics in the injured groups were due to increases in lung elastance (E) and resistance (R) (ΔE from pre‐injury = 46 ± 14 cmH_2_O L^−1^, *p *< 0.0001; ΔR from pre‐injury: 3 ± 2 cmH_2_O L^−1^ s^−^, *p *= 0.30) rather than changes to the chest wall (ΔE from pre‐injury: 0.7 ± 1.6 cmH_2_O L^−1^, *p *= 0.99; ΔR from pre‐injury: 0.6 ± 0.1 cmH_2_O L^−1^ s^−^, *p *= 0.01). Both control groups had no change in respiratory mechanics. In conclusion, therapeutic hypothermia can reduce markers of injury and inflammation associated with experimentally induced short‐term ARDS.

## Graphical abstract

We sought to determine if therapeutic hypothermia was effective at reducing injury and inflammation in a porcine model of acute respiratory distress syndrome. Injury score was determined by the presence of alveolar fibrin deposition, alveolar inflammatory cell infiltration, and interstitial and intra‐alveolar edema. Injury score was significantly lower in the hypothermic injured group relative to the normothermic injured group.

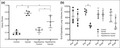

## 1 INTRODUCTION

Acute respiratory distress syndrome (ARDS) is a lung injury characterized by noncardiogenic pulmonary edema and rapid‐onset of severe hypoxic respiratory failure (Piantadosi & Schwartz, 2004; Ranieri et al., 2012). Despite efforts to improve outcomes, a recent multinational observational study found hospital mortality rates from ARDS of 40% (Zambon & Vincent, 2008). While the causes of ARDS are numerous, diffuse alveolar damage and neutrophil recruitment to the lungs are frequently observed (Belperio et al., 2006; Donnelly et al., 1996; Parsons et al., 2005). The subsequent release of toxic mediators damages the capillary endothelium and alveolar epithelium, allows the accumulation of proteinaceous edema in the interstitium and alveoli (Matthay & Zemans, 2011; Windsor et al., 1993), and precipitates further injury (Bevilacqua et al., 1985; Matthay & Zemans, 2011; Scheiermann et al., 2010; Williams et al., 2011; Zimmerman et al., 1999). Among its many deleterious effects, ARDS has a substantial influence on respiratory system mechanics (Henderson et al., 2017). The elastance and resistance of the respiratory system are characteristically increased, which can be largely attributed to changes in the physical properties of the lungs (Henderson et al., 2014). The changes in lung mechanics make mechanical ventilation challenging, as patients with ARDS have select regions of the lung that are collapsed while others well ventilated. As a consequence, these individuals have a reduced functional tidal volume (Gattinoni et al., 1987) and heterogeneous lung emptying (Pelosi et al., 1996). Mechanical ventilation is often required to support oxygenation in ARDS (Henderson & Sheel, 2012; Pinhu et al., 2003).

Therapeutic hypothermia used as an intervention in human studies suggests an improvement in neurological outcomes and a reduction in mortality following traumatic brain injury (Crompton et al., 2016) as well as ischemic brain injury (Ma et al., 2012; Orrock et al., 2016; Zhao et al., 2018) and has been used to decrease end‐organ injury in some surgical procedures (Kayatta & Chen, 2016). The purported mechanisms that underpin the benefits of therapeutic hypothermia include improvement of the ratio between oxygen supply and demand in at‐risk tissue, decreases in free radical formation, decreased release of pro‐inflammatory cytokines, and decreased polymorphonuclear leukocyte adhesion (Sinclair & Andrews, 2010). A modest number of small animal models (primarily rodents) of ARDS and ventilator‐associated lung injury (VALI) suggest that therapeutic hypothermia may improve the pulmonary mechanical and inflammatory derangements seen in ARDS (Huang et al., 2006; Lim et al., 2003; Shoji et al., 2005), presumably due to a reduction in inflammation (Lyden et al., 2005; Steinberg et al., 2004). Unfortunately, therapeutic hypothermia may not be as effective in treating select types of ARDS, as there is limited evidence suggesting it is futile in attenuating sepsis‐associated ARDS progression (Itenov et al., 2018). The ability to draw strong conclusions regarding the consequences of ARDS are limited in humans as many measurements are invasive. As such, most ARDS studies have been conducted on small animal models which do not provide an accurate representation of human pathophysiology; however, utilizing porcine models are beneficial as the respiratory anatomy and physiology of pigs are very similar to that of humans (Judge et al., 2014). In sum, the effects of hypothermia on alterations in pulmonary mechanics and inflammation in ARDS remain incompletely understood (Villar & Slutsky, 1993).

Unique to our study is the combination of physiologic, histologic, cytokine, and metabolomics measures to examine the influence of therapeutic hypothermia on lung injury. We examined the cellular mechanisms responsible for ARDS progression and integrated its consequences to a system level to further understand how ARDS impacts the body as a whole. The purpose of this study was to evaluate the utility of therapeutic hypothermia on the development of injury and inflammation in a porcine model of short‐term ARDS. We hypothesized that therapeutic hypothermia initiated simultaneously with injury, but achieved after the onset of lung injury would: (i) mitigate the increase in elastance and resistance of the respiratory system associated with ARDS, (ii) decrease the serum cytokine and metabolomics changes seen in experimental ARDS, and (iii) decrease the inflammatory histologic and physiologic changes observed in lung tissue following the induction of experimental ARDS.

## 2 METHODS

### 2.1 Animals and instrumentation

**FIGURE 1:**
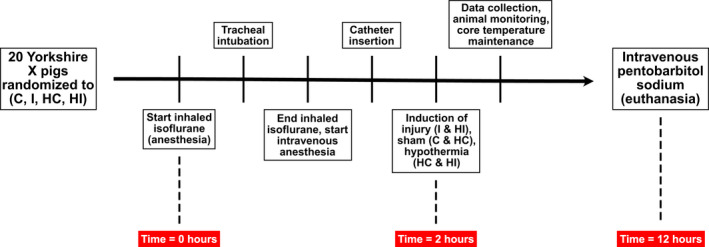
Outline of the sequence of methods employed in the present study. Pigs were randomized into four groups: control (C), injured (I), hypothermic control (HC), and hypothermic injured (HI)

The Animal Research Committee of the University of British Columbia (certificate # A12‐0230) reviewed and approved the experimental procedures. Twenty adult female Yorkshire X pigs (~14 weeks old) were divided into four equal groups: control (C), injured (I), hypothermic control (HC), and hypothermic injured (HI). The sequence of methods used for each animal in the present study is represented in Figure 1.

Inhaled isoflurane (3–5%, in oxygen) was used to induce anesthesia. After tracheal intubation, intravenous anesthesia (midazolam: 0.1 mg kg^−1^ intravenous and propofol infusion: 200 μg kg^−1^ min^−1^ and adjusted to between 150 and 300 μg kg^−1^ min^−1^) was started and inhalational anesthesia discontinued. A catheter was placed in the right femoral artery to measure blood pressure and collect blood samples into pre‐heparinized syringes. Blood gases were analyzed by a calibrated blood gas analyzer (ABL 80 CO‐OX Flex, Radiometer, Copenhagen, Denmark) and were corrected offline for core temperature. Mean arterial blood pressure was maintained >60 mmHg with an infusion of phenylephrine at 0.1–0.3 μg kg^−1^ min^−1^ intravenously when required. A pulmonary artery catheter was placed via the right jugular vein and cardiac output was measured using a bolus thermodilution method. A urinary catheter and rectal temperature probe were placed. All animals received approximately 1081 ± 208 ml of intravenous fluids.

All animals had a cooling catheter (Zoll Thermoguard XP, Asahi Kasei Group, Japan) placed through the left jugular vein to continuously measure core temperature and control body temperature (Henderson et al., 2015; Luecke et al., 2006). The catheters use cold fluid circulating in a closed circuit from an external refrigerator/pump to allow rapid cooling (see below).

Mechanical ventilation (Puritan‐Bennett 7200, Covidien, Ireland) was provided with an inspired oxygen fraction (FiO_2_) of 0.5 and an inspiratory flow of 60 L min^−1^. The ventilator settings were based on standard of care in humans with ARDS (Acute Respiratory Distress Syndrome Network, 2000). Specifically, all animals initially received a tidal volume of 6 cc kg^−1^ of body weight. If end‐inspiratory pressure exceeded 30 cmH_2_O, tidal volume was decreased to a minimum of 4 cc kg^−1^. To minimize interanimal heterogeneity in lung recruitment, a fixed positive end‐expiratory pressure of 5 cmH_2_O was maintained. Respiratory rate was initiated at 15 breaths·min^−1^ and adjusted to maintain serum pH > 7.30.

### 2.2 Induction of lung injury

In the I and HI groups, oleic acid was infused through the pulmonary arterial catheter in a series of 0.1 ml injections until the arterial oxygen tension to inspired oxygen fraction ratio (P/F ratio) was decreased and a plateau in airway pressure was observed (Grotjohan et al., 1996). The animals in the I group and the HI group received a total of 0.69 ± 0.26 ml and 1.02 ± 0.46 ml of oleic acid, respectively. The point where the P/F ratio decreased and a plateau in airway pressure observed was denoted as time = 2 h with the beginning of the experiment (induction of anesthesia) and baseline being denoted as time = 0 h. The control animals (C and HC) received a sham injection of similar volume of saline 2 h after the beginning of the experiment (time = 2 h). Oleic acid infusion induced early mortality in three animals due to shock, as such, the data from these animals has not been included.

### 2.3 Intervention

Normothermic animals (groups C and I) had core temperatures maintained between 36–38.5°C throughout the experiment. Prior to time = 2 h or injury, the hypothermic animals (groups HC and HI, respectively) had core temperatures maintained at 36–38.5°C. Core temperature in the HC and HI groups was lowered to 32°C within 60 min following time = 2 h, such that the target core temperature was reached approximately 45 min after the injury was achieved. Temperature was maintained for ~10 h (36–38.5°C in I and C or 32°C in HI and HC), after which euthanasia was achieved using pentobarbital sodium (120 mg/kg intravenous). Death was confirmed by the absence of a pulse and cardiac electrical activity.

### 2.4 Measurement of pulmonary mechanics

Flow and pressure data were sampled and recorded digitally (PowerLab/16SP model ML 795 and Chart v7, ADI, Colorado Springs, CO). Inspiratory and expiratory flows were measured using two separate calibrated pneumotachographs (Model 3813, Hans Rudolph, Kansas City, MO). Airway pressure was measured through a wye in the ventilator circuit near the mouth and was connected to a calibrated pressure transducer (model 1110, Hans Rudolph, Kansas City, MO). Esophageal pressure was measured using a balloon‐tipped catheter (no. 47‐9005, Ackrad Laboratory, Cranford, NJ) placed in the lower third of the esophagus. The distal end of the catheter was connected to a similar calibrated pressure transducer. Catheter position was deemed satisfactory if the change in the esophageal pressure was equal to the change in airway pressure during passive inspiration (Talmor et al., 2006). Pulmonary mechanics of the respiratory system and lung was calculated using previously described methods before and after injury (Luecke et al., 2006). End‐expiratory lung volume was measured via the helium dilution technique (Henderson et al., 2015).

### 2.5 Serum and bronchoalveolar lavage assays

All animals had blood samples for complete blood count and arterial blood gas analysis as well as serum samples for cytokine assays and metabolite determination drawn prior to lung injury at time = 0 h (baseline) and 4, 8, and 12 h (post injury). Additionally, all animals underwent bronchoalveolar lavage (BAL) immediately before the lung injury intervention and 4, 8, and 12 h after baseline using a modification of previously described BAL procedures (Ganter & Hensel,). Direct cellular counts were performed on an aliquot of BAL fluid by an automated cell counter. Direct and cytocentrifuge smears were prepared for cytology examination using modified Wright's Giemsa stain and interpreted by a laboratory unrelated to the study.

### 2.6 Light microscopy

Representative samples of lung were harvested from the right diaphragmatic lobe and submersion fixed in 10% buffered formalin using previously described techniques (Matute‐Bello et al., 2011). Fixed tissues were processed on a Tissue‐Tek VIP 5 Vacuum Infiltration Tissue Processor (Sakura Finetek USA, California, USA). For each animal, twenty randomly selected fields were assessed at 400× magnification. A veterinary pathologist who was not involved in the study and was blinded to animal assignments, reviewed the slides and assigned a composite histologic injury score to each animal based on: (i) alveolar fibrin deposition; (ii) alveolar inflammatory cell infiltration; and (iii) interstitial and intra‐alveolar edema (Kao et al., 2004).

### 2.7 ^1^H‐NMR spectroscopy analysis and metabolite concentration profiling

One dimensional proton nuclear magnetic resonance (^1^H‐NMR) spectroscopy analysis was performed using a 600 MHz Bruker Ultrashield Plus NMR spectrometer (Bruker BioSpin Ltd., Canada). We used ChenomX NMR Suite 7.1 software (ChenomX Inc., Edmonton, Alberta, Canada) for profiling NMR spectra to identify and quantify metabolites in a non‐targeted approach (Weljie et al., 2006). DSS (4,4‐dimethyl‐4‐silapentane‐1‐sulfonic acid) was added to samples as an internal standard (Wishart et al., 2009). Two hundred microliters of plasma was filtered using a 5 times prewashed 3 KDa filter (NanoSep microcentrifuge) at 12,000 × g for 1 h at 4ºC followed by a rinse using 100 μl D_2_O. The filtrates were adjusted to 400 μl by adding 80 µl of phosphate buffer (0.5 M NaH_2_PO_4_ buffer solution at pH 7.0) containing 2.5 mM 2,2‐dimethylsilapentane‐5‐sulfonate (DSS, final concentration 0.5 mM), 10 µl sodium azide (1 M NaN_3_), and D_2_O. All samples were adjusted for pH 7.0 ± 0.04 at room temperature.

### 2.8 Metabolomic data acquisition

NMR data were generated on samples run in a randomly and blinded fashion using a cooled (4°C) automated sample changer on the NMR spectrometer. The one‐dimensional NMR spectra were obtained using 1D proton spectroscopy pre‐saturation pulse sequence (noesyppr 1d) to optimize water suppression and a mixing time of 100 ms (Li et al., 2016; Qiu et al., 2019) using 1024 scans, zero filled and Fourier transformed to 128k points. All spectra were corrected by phasing, broadening and baseline correction based on the DSS peak at 0.0 pm using Topspin software program (Bruker BioSpin Ltd., Canada).

### 2.9 Metabolite concentration profiling

NMR spectra were analyzed to identify and quantify metabolites using the ChenomX NMR Suite 7.1 software (ChenomX Inc., Edmonton, Alberta, Canada) in a non‐targeted profiling approach. All spectra were phased and followed by baseline correction and water region deletion using a processor module (Weljie et al., 2006). All spectra were profiled to measure metabolite concentration using the DSS standard concentration to quantify each metabolite using the profiler module. Metabolite concentrations were normalized to the median of fold change used for statistical data analysis.

### 2.10 Metabolomic data analysis

We applied multivariate and univariate data analyses to extract information from metabolomics data using SIMCA‐P (Version 15.0.2 Umetrics AB, Umea, Sweden) and MetaboAnalyst 5 software, respectively. Obtained normalized data were used after log transformation and univariance scaling or autoscaling for multivariate and univariate analyses.

### 2.11 Multivariate data analyses

Unsupervised multivariate principal component analysis was performed to assess the obtained data on plasma ^1^H‐NMR metabolites to observe intrinsic relationship between samples, trends, similarities, and outliers based on the identified metabolites by NMR (*n* = 54 metabolites). Principal component analysis models were obtained using a five‐component method to summarize the information in the NMR‐based metabolomics dataset derived from all plasma samples.

Partial least‐squares discriminant analysis using regression and prediction methods was applied to show the separation between all four groups at the same time. Also, orthogonal partial least‐squares discriminant analysis was performed to show the maximum separation between two groups (predictive classifications) and finding which variables are responsible for the discrimination. Three parameters, CV‐ANOVA *p* value, R^2^Y, and Q^2^Y, were considered to verify the quality of the prediction models obtained by partial least‐squares discriminant analysis and orthogonal partial least‐squares discriminant analysis. In addition, sensitivity, specificity, and area under the receiver operating curve were calculated to describe the predictive models. The predictive models were created based on the most important variables that were obtained using variable importance in projection scores (≥1.0) which are estimated by the importance of each variable in the models.

### 2.12 The prediction set modeling

We built a prediction model for misclassification test to obtain sensitivity, specificity, and area under the receiver operating curve for partial least‐squares discriminant analysis and orthogonal partial least‐squares discriminant analysis models. This process was repeated three times for randomly creating prediction models and averaged sensitivity and specificity were considered over three runs.

### 2.13 Univariate data analysis

We applied univariate analysis to enhance the obtained information from ^1^H‐NMR metabolomics data on plasma samples as a less complicated method to understand the group differences. MetaboAnalyst 5.0 software was used for univariate analysis including *T*‐test for evaluation of the difference of each variable individually between the two groups and ANOVA to show the variable changes among all groups at the same time. The important metabolites were selected based on the *t*‐test and false discovery rate with the threshold being ≤0.05. Additionally, the heatmap visualizations were created using MetaboAnalyst 5.0.

### 2.14 Cytokine profiling

Millliplex^®^, Bio‐Plex^®^, or Procartaplex™ kits (affymetrix eBioscience) coupled with 96‐well plates were used for cytokine profiling. Multiplex Luminex immunoassay analysis was performed to measure nine cytokines including interferon‐alpha (IFN‐α), interferon‐gamma (IFN‐γ), interleukin‐1beta (IL‐1β), IL‐10, IL‐4, IL‐6, IL‐8, IL‐12, and tumor necrosis factor (TNF)‐α. For immunoassay analysis, 25 μl of frozen plasma samples were thawed on ice and mixed well by vortexing followed by centrifugation at 10,000 × g for 5–10 min to remove particulates before running the multiplex protocol.

### 2.15 Statistics

Values are displayed as mean ± standard deviation (SD). Physiological variables and pulmonary mechanical data were analyzed using a three‐way ANOVA and Tukey's correction for multiple comparisons analysis. Injury score analysis was performed using a two‐way ANOVA and Tukey's correction for multiple comparisons analysis. Statistical significance was defined at *p *< 0.05. Statistical analyses were performed using GraphPad Software (Version 8.2.1, La Jolla, CA, USA). Metabolomic analysis was performed using a two‐way ANOVA where appropriate. Statistical analyses were performed using STATA 10.0 Statistical Software (StataCorp, College Station, TX) and SAS (SAS Institute, Inc., NC). Metabolomics statistical analysis was described above.

## 3 RESULTS

### 3.1 Descriptors, temperature management, and oxygenation

**FIGURE 2:**
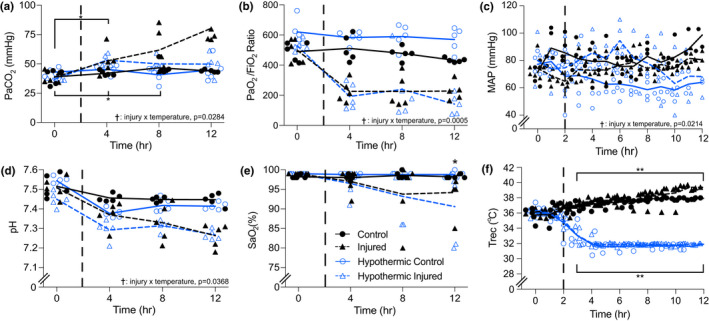
Physiologic variables measured over 12 h in normothermic control and injured and hypothermic control and injured groups. Dashed vertical lines within each graph represent induction of injury (2 h). Panel (a) * injury by time interaction (*p *= 0.01) in the C group between 0 and 4 h and in the I group between 0 to 4 h and 0 to 8 h. † injury by temperature interaction (*p *= 0.0284) in the C versus I groups. (b) * injury by time interaction (*p *= 0.0039) in the C versus I groups at 4, 8, and 12 h. † injury by temperature interaction (*p *= 0.0005) in C versus I, C versus HI, HC versus I, and HC versus HI groups. (c) † injury by temperature interaction (*p *= 0.0214) in the C versus HC groups. (d) † injury by temperature interaction (*p *= 0.0368) in C versus I groups (*p *= 0.0058) and C versus HI groups (*p *= 0.0017). (e) * injury by time interaction (*p *= 0.0001) at 12 h in C versus I groups. *, significant interaction of injury by time. **, significant interaction of temperature by time. †, significant interaction of injury by temperature. All individual data points are presented, lines indicate averages

The mean body mass of all animals was 50.0 ± 6.3 kg. The C, I, HC, and HI subjects weighed: 45.9 ± 9.3 kg, 53.0 ± 3.4 kg, 53.4 ± 3.3 kg, and 47.9 ± 5.1 kg, respectively, with no difference between groups (*p *= 0.13). Respiratory rates were 20.8 ± 5.6 breaths min^−1^, 19.0 ± 2.8 breaths min^−1^, 15.2 ± 3.7 breaths min^−1^, and 10.6 ± 0.3 breaths min^−1^ for the C, I, HI, and HC, respectively. Respiratory rates were significantly different between the C and HC groups (*p *= 0.002) and the I and HC group (*p *= 0.01). Goal temperature was reached in all four groups within 60 min of time = 2 h (Figure 2f). There was no difference in rectal temperature between all groups prior to injury induction (*p *= 0.28); however, from the time of injury onwards, the hypothermic groups had a significantly lower rectal temperature relative to the normothermic groups (*p *< 0.0001). No adverse events were associated with the insertion of cooling catheters or with the induction and maintenance of hypothermia.

Cardiac output did not differ between groups before or after injury/sham (*p *= 0.31; C pre = 6.04 ± 2.07, post = 5.25 ± 1.67 L min^−1^; I pre = 4.95 ± 1.06, post = 5.26 ± 1.44 L min^−1^; HC pre = 5.41 ± 1.85, post = 5.38 ± 2.92 L min^−1^; HI pre = 5.15 ± 1.32, post = 4.06 ± 1.23 L min^−1^). Mean arterial pressure (MAP) was maintained at approximately 60 mmHg in all groups both before and after injury (Figure 2c). Animals in the I and HI groups were given an infusion of phenylephrine (31.14 ± 11.17 ml and 70 ± 12.24 ml; *p *= 0.0008, respectively) to maintain MAP during sedation.

### 3.2 Arterial blood gases and core temperature

Arterial blood gases and core temperature are presented in Figure 2. Arterial partial pressure of carbon dioxide did not differ between most of the groups prior to or during injury (C, HC, and HI), but gradually increased in the I group throughout data collection (Figure 2a). The P/F ratio remained consistent in the injured groups prior to and following injury (I and HI) but was significantly lower relative to the control groups (C and HC) (Figure 2b). Following induction of injury, the pH was found to be significantly lower in the injured groups compared to the control groups (*p *= 0.0368; Figure 2d). Arterial oxygen saturation (SaO_2_) in the C and HC groups did not change throughout the experiment and was significantly greater than the I and HI groups at all time points (*p *= 0.0048; Figure 2e). The SaO_2_ in the I and HI groups progressively decreased following injury induction, with the HI group SaO_2_ being substantially less than that of the I group at approximately 12 h.

### 3.3 Pulmonary mechanics

**FIGURE 3:**
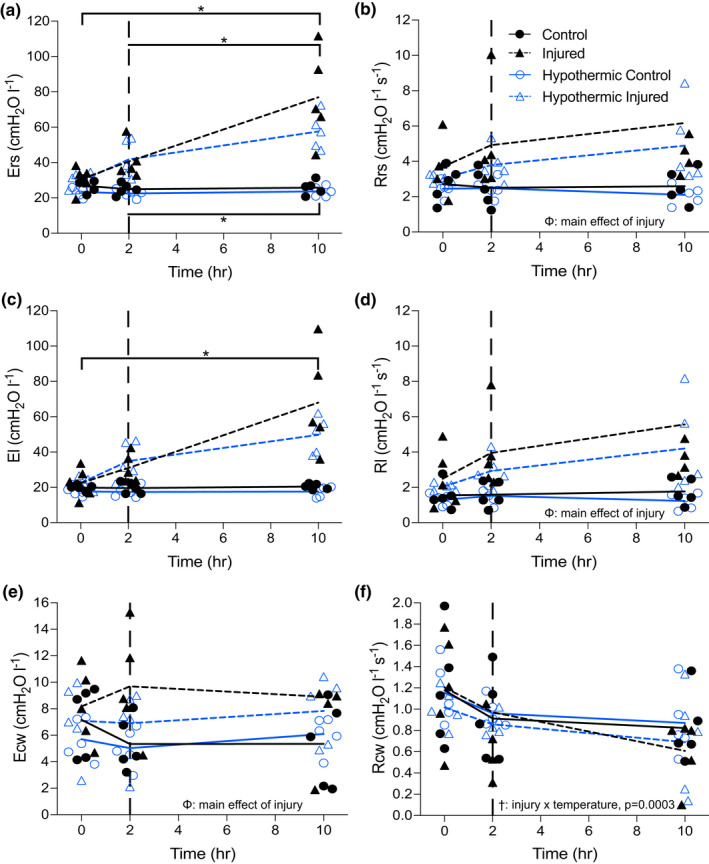
Elastance (E; Panels a, c, e) and resistance (R; Panels b, d, f) of the respiratory system (rs; Panels a, b), lung (l; Panels c, d), and the chest wall (cw; Panels e, f) measured over 10 h in the four groups. Dashed lines within each graph represent induction of injury (2 h). All individual data points are presented, lines indicate averages. Panel (B) Φ main effect of injury (*p *= 0.0007). (d) Φ main effect of injury (*p *= 0.0006). (e) Φ main effect of injury (*p *= 0.0365). (f) injury by temperature interaction (*p *= 0.0003). *, significant interaction of injury by time. †, significant interaction of injury by temperature. Φ, significant main effect of injury

Pulmonary mechanics data are presented in Figure 3. Elastance of the respiratory system (Ers) (Figure 3a) and of the lung (El) (Figure 3c) were significantly greater in the I and HI groups following injury compared to baseline (Ers, *p *= 0.006; El, *p *= 0.0124) and compared to the C and HC groups (Ers, *p *= 0.0056; El, *p *= 0.0128). Ers was greater in the I compared to the HI group at 10 h following injury induction; however no significant difference was observed. The rise in Ers was due to changes in the El since there were no significant changes in chest wall elastance (Ecw) following injury (*p *> 0.99; Figure 3e). There was no difference in Ers, El, or Ecw in the control groups (C and HC). There were no differences in the respiratory system resistance (Rrs), lung resistance (Rl), and chest wall resistance (Rcw) between all groups prior to injury. Following injury, Rrs increased in the injured groups compared to controls, due to increased Rl. There were no significant differences observed in Rcw between the groups at any time point (*p *= 0.8513).

**FIGURE 4:**
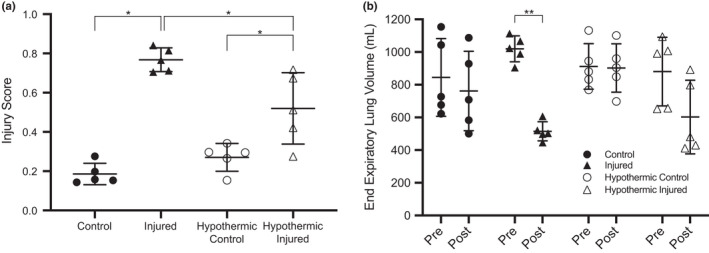
Injury score (Panel a) and end expiratory lung volume (EELV) (Panel b) measured in normothermic control, normothermic injured, hypothermic control, and hypothermic injured groups. Injury score based on three variables: (i) alveolar fibrin deposition, (ii) alveolar inflammatory cell infiltration, and (iii) interstitial and intra‐alveolar edema. All individual data points are presented, horizontal lines represent the median while the error bars are 95% confidence intervals. *, significant interaction of injury by temperature (*p *< 0.05), ** significant difference between pre‐injury and post‐injury EELV (*p *< 0.05)

End‐expiratory lung volume decreased from 1020 ± 79 ml pre‐injury to 515 ± 59 ml post injury in the I group (*p *= 0.0025) (Figure 4b **)**. Whereas EELV was not different in the HI group before and after injury (912 ± 210 ml vs. 903 ± 225 ml). No significant differences in EELV were observed in the control groups.

### 3.4 Histology

**FIGURE 5:**
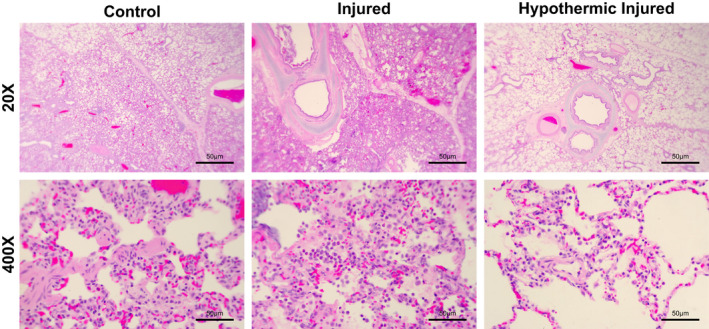
Representative samples of the lung harvested from the right diaphragmatic lobe. Histology of lung tissue from the normothermic control, normothermic injured, and hypothermic injured groups. Control images display normal alveolar structure with some neutrophils present in the interstitial space. Injured images display neutrophil infiltration in both the alveolar and interstitial space, hyaline membrane formation, alveolar septal thickening, and proteinaceous debris filling the airways. Hypothermic injured images display both neutrophil infiltration in the alveolar and interstitial space with some proteinaceous debris present, yet no hyaline membrane formation or alveolar septal thickening. Alveolar tissue integrity preserved to an extent in the HI group with considerably less inflammatory activity present

Injury scores based on an objective three‐tiered lung injury scoring system (Matute‐Bello et al., 2011) are displayed in Figure 4a and representative photomicrographs are shown in Figure 5. The lung injury scoring system was selected for two reasons: (i) it is specific to animal lung injury, and (ii) it is consistent with previously published literature (Matute‐Bello et al., 2011) thus facilitating between‐study comparisons; however, it is important to note that there is currently no agreed‐upon diagnostic criteria for animal lung injury. Lung injury score was based on five factors: (A) neutrophils in the alveolar space, (B) neutrophils in the interstitial space, (C) hyaline membranes, (D) proteinaceous debris filling the airspaces, and (E) alveolar septal thickening (Table S1). Relative to C, the I animals displayed a larger injury score (*p *< 0.0001). The injury score was significantly lower in the HI group compared to the I group (*p *= 0.0115) and the HI were significantly greater than HC (*p *= 0.0111). There were no significant differences observed between the C and HC groups.

Light microscopy examination of the lungs of the C and HC groups revealed minimal neutrophil infiltrate in the alveolar and interstitial space, little to no inflammation present with some alveolar septal thickening (Figure 5a). Conversely, the animals in the I group displayed considerable neutrophil infiltrate in the interstitial and alveolar spaces, proteinaceous debris, hyaline membrane formation in and around the alveoli as well as alveolar septal thickening (Figure 5b). Likewise, the animals in the HI group had a similar phenotype to that of the I group, yet there was no hyaline membrane formation in 4/5 animals with very little proteinaceous debris and alveolar septal thickening compared to that of the I group (Figure 5c).

### 3.5 White blood cell responses

**FIGURE 6:**
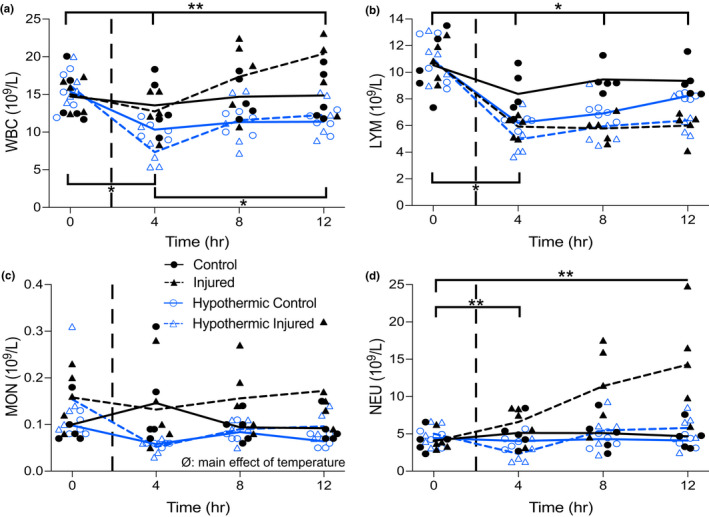
Bronchoalveolar lavage fluid data measured over 12 h in the normothermic control and injured and hypothermic control and injured groups. Dashed vertical lines within each graph represent induction of injury (2 h). Panel (a) ** temperature by time interaction (*p *= 0.0002) in the hypothermic groups between 0 to 4 h and 0 to 12 h. * injury by time interaction (*p *= 0.0072) in the injured groups at 0 to 4 h and 4 to 12 h. Panel (B) * injury by time interaction (*p *= 0.0001) in the control groups at 0 to 4 h and in the injured groups between 0, 4, 8 and 12 h. Panel (c) ∅ main effect of temperature (*p *= 0.0358). Panel (d) ** temperature by time interaction (*p *= 0.0066) in the normothermic groups at 0 to 12 h and in the hypothermic groups at 0 to 4 h. *, significant interaction of injury by time. **, significant interaction of temperature by time. ∅, significant main effect of temperature. All individual data points are presented, lines indicate averages

BAL fluid data are presented in Figure 6 and display the BAL cell counts of white blood cells (WBC), lymphocytes, monocytes, and neutrophils. BAL fluid WBC count did not differ between groups prior to injury; however, WBC count gradually increased in the I group with the progression of ARDS. Additionally, it was observed that hypothermia effects WBC concentration as both hypothermic groups (HC and HI) had less WBCs compared to the normothermic groups (C and I) (*p *= 0.0052) (Figure 6a). BAL fluid lymphocyte count was not different across groups prior to injury but count decreased below baseline in all groups with the injured groups (I and HI) displaying the lowest concentrations (Figure 6b). BAL fluid monocyte count differed prior to injury between the control (C and HC) and injured groups (I and HI). The monocyte concentrations in the control groups appeared lower than that of the injured groups prior to injury induction. Following injury induction, monocyte concentration increased in the I group with ARDS progression with the rest of the groups (C, HC and HI) having lesser concentrations, yet this observation was not significant (*p *= 0.1095) (Figure 6c). Lastly, BAL fluid neutrophil count was the same between the groups prior to injury induction. Neutrophil concentrations were not different between the C, HC, and HI groups during ARDS progression; however, there was a significant increase in neutrophil content in the I group overtime (*p *= 0.0315) (Figure 6d).

### 3.6 Metabolomics

The induction of lung injury and hypothermia significantly altered the metabolic and metabolomic profiles across the groups. In terms of metabolite concentrations, both multivariate and univariate analysis showed that the I group was significantly different from that of the C group. Orthogonal partial least‐squares discriminant analysis demonstrated that 26 out of 54 metabolites contributed to the highly predictable (Q2 = 0.4) and significant (*p *= 0.00029) separation of the I and C groups (Figure S1). The metabolites with the largest differentiation between the I and C groups included increased concentrations of fumarate, acetone, 3‐hydroxybutyrate, isobutyrate, and acetoacetate. The metabolites that decreased the most in the I group compared to the C group included 3‐hydroxyisovalerate, isoleucine, valine, arginine, tyrosine, and asparagine (Figure S2 and Table S5). Additionally, univariate analysis revealed a similar trend of metabolite changes (Table S2). Moreover, there was a significant change in metabolite profiles in the I group relative to the C group over time (Figure S3).

Hypothermia had a significant effect on the metabolic and metabolomic profiles of the I and C groups, with corresponding significant alterations in the metabolic and metabolomic profiles between the HI and I groups as well as the HC and C groups (Figures S4 and S7). The HI and I groups can be separated in a remarkably predictable (Q^2^ = 0.64) and significant manner (Figure S4). The metabolites that increased the most in the HI relative to I group include acetate, isoleucine, 3‐hydroxyisovalerate and arginine. The metabolites that decreased the most in the HI relative to I group include succinate, methionine, 4‐hydroxybutyrate, and β‐alanine. Additionally, the univariate t‐test analysis verified the findings of the multivariate analysis with respect to the significant changes observed in the metabolites. There were 20 out of 54 metabolites that were significantly different between the HI and I groups (all *p *< 0.05; Table S3). The metabolite changes remained consistent over time in the HI group compared to that of the I group, which had a greater degree of fluctuation in metabolite concentrations (Figure S6).

Additional metabolomic analyses revealed that the HC and C groups expressed substantially different metabolites and metabolomic profiles. The separation of the HC and C groups were predictable (Q^2 ^= 0.8) and significant (*p *< 0.001) (Figure S7). When considering the HC and C groups, the metabolites that increased the most with hypothermia included formate, ornithine, histidine, glucose, and mannose while the metabolites that decreased the most due to hypothermia included arginine, succinate, and asparagine, (Figure S8 and Table S5). The metabolite alterations for both the HC group and I group were consistent over time (Figure S9). Table 1 shows the characterization of separation models based on the most differentiating metabolites and cytokines with extreme sensitivity, specificity, and area under the curve for all models.

**TABLE 1:** Characterization of OPLS‐DA discrimination models to separate groups based on the most differentiating metabolites and cytokines

Separation model	R2Y	Q2Y	*p* value	Sensitivity	Specificity	AUC	# metabolites/cytokine
Metabolomics: I vs. C	0.62	0.4	0.00029	100	100	0.92	26
Cytokine: I vs. C	0.1	0.072	0.2	—	—	—	—
Metabolomics: HI vs. I	0.785	0.64	2.1e‐10	93	95	1.00	20
Cytokine: HI vs. I	0.578	0.432	1.02e‐05	95	64	0.84	9
Metabolomics: HC vs. C	0.868	0.79	3.2e‐10	100	100	1.0	26
Cytokine: HC vs. C	0.829	0.778	3.7e‐09	90	100	0.95	8

Abbreviations

I, injured; C, control; HI, hypothermic injured; HC, hypothermic control.

### 3.7 Cytokines

**FIGURE 7:**
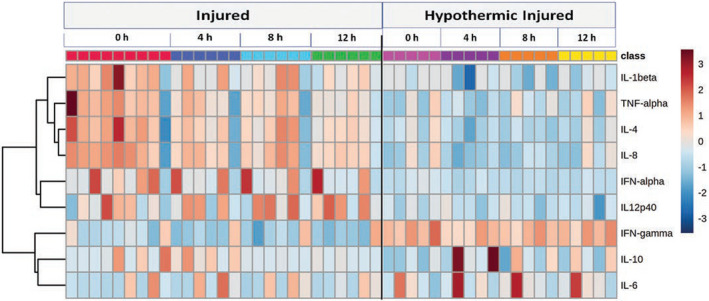
Heatmap analysis reveals the change in metabolites overtime in the I and HI groups. Metabolite changes are remarkably higher in the HI group compared to the I group

The I and C groups were not significantly different in terms of cytokines since the discrimination model was not predictive (Q^2^ = 0.072) or significant (*p *= 0.20) and none of the cytokines significantly changed between the I and C groups (Table 1). Nonetheless, within‐group comparison of the I group showed a significant difference between baseline (time = 0 h) and each time point (time = 4, 8, 12 h post baseline). IL‐12 significantly increased at 4, 8, and 12 h following baseline measures when compared to baseline (time = 0 h) (Figure S10). Discrimination models revealed that cytokine profiles in the HI and HC groups were significantly different from the I and C groups, respectively, in predictable and significant manners. The separation of the HC and C groups was more predictive than the separation of the HI and I groups (Q^2^ = 0.74 vs. 0.43; Table 1). Analyses showed that most of the pro‐inflammatory cytokines, except for IFN‐γ, decreased in the HI group compared to the I group. Additionally, an increase in IL‐4, IFN‐α and IL‐10 and a decrease in IFN‐γ and TNF‐α were observed in the HC group compared to the C group (Figure 7 and Figure S11). Table S4 shows the characterization of separation models based on the most differentiating cytokines that is comparable with the metabolic results for the separation of both the HC and C groups and the HI and I groups (Table 1). Therapeutic hypothermia significantly changed the cytokine profiles between the HC and C groups; however, these changes were less apparent between the HI and I groups compared to that of the metabolite and metabolomic alterations.

## 4 DISCUSSION

### 4.1 Major findings

The major findings from this study are twofold. First, therapeutic hypothermia reduced lung injury score and white blood cell counts suggesting that hypothermia was able to reduce the production and alveolar infiltration of pro‐inflammatory cells associated with injury in ARDS. Second, all groups expressed unique metabolomic phenotypes with the HI group producing less pro‐inflammatory metabolites that facilitate energy production such as lactate, succinate, β‐alanine, methionine, fumarate, and acetoacetate (Chen et al., 2016; Gonçalves‐de‐Albuquerque et al., 2015; Hope et al., 2021; Niu et al., 2012; Peiró et al., 2016). This suggests therapeutic hypothermia was effective in reducing the production of pro‐inflammatory metabolites responsible for injury generation in ARDS. Overall, our results suggest that therapeutic hypothermia was effective in reducing some markers of injury and inflammation in a porcine model of experimental ARDS.

### 4.2 Histology and injury score

By design, intrapulmonary injection of oleic acid led to the development of mechanical and physiological alterations like that of ARDS in humans. Oleic acid initiates the production of pro‐inflammatory signalling molecules (Gonçalves‐de‐Albuquerque et al., 2015; Weljie et al., 2006) that cause severe inflammatory lung injury consistent with ARDS (Gonçalves‐de‐Albuquerque et al., 2015), such as increases in alveolar epithelial and capillary endothelial permeability as well as alternations in gas exchange and lung mechanics (Matthay et al., 2019). The histological findings revealed that therapeutic hypothermia was effective in reducing injury and inflammation (i.e., alveolitis) in experimental ARDS. As previously mentioned, the HI group received more oleic acid compared to the I group (*p *= 0.1989) to achieve a similar P/F ratio and the HI group still demonstrated an attenuated injury response. As such, the attenuation of injury in the HI group is likely not the result of a lower dose of oleic acid. We found that the HI group had a lower injury score compared to that of the I group (Figure 4a). Additionally, we found that WBC, monocyte, and neutrophil counts were reduced in the HI compared to I group, with no change observed in lymphocyte count (Figure 6a, c–d). Furthermore, we found that therapeutic hypothermia was effective in reducing the production of pro‐inflammatory cells in the HI group compared to the I group (Figure 6). Our findings are consistent with the proposed mechanism of therapeutic hypothermia as it reduces pro‐inflammatory pathway and cell production (Sinclair & Andrews, 2010). Lymphocyte count may remain unchanged with therapeutic hypothermia and/or injury as hypothermia can reduce lymphocyte count (Bouma et al., 2011) and severe lung injury may destroy lymphocytes entirely (Venet et al., 2009; Verjans et al., 2018); however, when lymphocyte counts are low, alveolar macrophages compensate for their reduction (Verjans et al., 2018). Additionally, lymphocyte cell counts observed below baseline in all groups could be attributed in part to sedation as all animals received similar amounts of sedation (~35–40 ml) and there is evidence suggesting lymphocyte counts can decrease when under general anesthesia (Liu et al., 2011). Moreover, since both monocytes and neutrophils contribute to lung injury and inflammation (Bouma et al., 2011; Jiang et al., 2020; Rosseau et al., 2000), the removal of these cells may impede lung injury progression associated with ARDS.

In the control groups (C and HC), therapeutic hypothermia induced a minimal reduction in WBCs, monocytes, lymphocytes, and neutrophils; however, these changes were not significant (Figure 6). Therapeutic hypothermia was effective in reducing injury likely by decreasing the migration and infiltration of leukocytes (i.e., neutrophils) into the alveolar space. In the C group, there is a baseline concentration of inflammatory cells present which is common as they are nearby to respond to injury if such situations arise (i.e., lung injury, ARDS) (Matthay et al., 2019). Therapeutic hypothermia can attenuate the expression of pro‐inflammatory pathways and cell production (Sinclair & Andrews, 2010), specifically, the infiltration and migration of pro‐inflammatory cells into the alveolar space (Matthay & Zemans, 2011). As such, we observed a reduction in WBCs, monocytes, and neutrophils in the HI group relative to I group.

### 4.3 Inflammatory metabolites and cytokines

Therapeutic hypothermia induced large changes in the metabolomic profile of the HI group compared to that of the I group. The metabolomic alterations observed in the present study are likely the result of experimentally induced ARDS rather than the oleic acid (lipid) infusion. While the oleic acid infusion may have induced secondary changes to the metabolomics data (i.e., energetics), the infusion would not have had any direct effects on the metabolites quantified in this study especially since the ^1^H‐NMR method used did not quantify lipids. We did, however, find an elevation of ketone bodies and aromatic amino acids, particularly tyrosine, in the I group, which may be related to increased immune activity (Yang et al., 2019). Ketogenic conditions can favor anti‐inflammatory pathways and neuroprotective effects while inducing inflammation (Miyamoto et al., 2019). Additionally, there is evidence to suggest that tryptophan and phenylalanine metabolism correlate highly with inflammation (Strasser et al., 2017). Moreover, increased amino acid metabolism, including energy metabolism, could be a response to elevated inflammatory activity (McGaha et al., 2012). Cytokines are well‐recognized proteins involved in immunity, whereas the involvement of metabolites are less well known. The metabolic phenotype response to injury induction was more significant than that of the cytokine response; however, injury induction caused considerable changes in concentration of both pro‐inflammatory and anti‐inflammatory markers. In the HI group, lactate, succinate, β‐alanine, methionine, fumarate, and acetoacetate decreased relative to the I group, which may be related to the reduction in inflammation (Chen et al., 2016; Gonçalves‐de‐Albuquerque et al., 2015; Hope et al., 2021; Niu et al., 2012; Peiró et al., 2016). Moreover, cytokine analysis revealed a reduction in inflammatory activities in the HI group as most of the pro‐inflammatory markers such as TNF‐α, interferon beta (IFN‐β), and IL‐8 decreased in the HI group compared to I group. In the HC group compared to the C group, increased histidine, ornithine, and glucose may exhibit anti‐inflammatory effects and inhibition of oxidative stress (Akinci et al., 2005; Chen et al., 2016; Dominelli & Sheel, 2012; Hope et al., 2021) and decreased arginine and asparagine could be associated with reduced inflammation (Bouma et al., 2011; Sahetya & Brower, 2017) that may imply the potent anti‐inflammatory property of therapeutic hypothermia on normal conditions. Among the cytokine analysis, both anti‐inflammatory and pro‐inflammatory markers increased and decreased in the HC group. Nonetheless, the HI and HC groups did not show similar metabolite alterations when compared to the I and C groups, respectively. As expected, the hypothermia effect could not be identical in the injured and control groups, since the hypothermia effect could partially compensate for the metabolite alterations of the pathological conditions to the baseline in the I group. Conversely, hypothermia could slow down all normal physiologic functions to lower points of baseline level in C groups. Overall, metabolic alterations following injury induction may be associated with elevated inflammation‐based metabolites, whereas therapeutic hypothermia may reduce inflammation‐based metabolites. We demonstrated that the metabolic response to injury and therapeutic hypothermia was significant and consistent compared to the cytokine response seen post‐injury induction. Moreover, the short‐term follow‐up post injury may contribute to the low cytokine response we observed.

### 4.4 Pulmonary mechanics and arterial blood gases

Increased alveolar and capillary permeability may facilitate the infiltration of proteins, blood, and fluid into the alveoli causing atelectasis. Additionally, increased alveolar epithelial cell death may lead to the formation of hyaline membranes and fibrin deposition, thereby reducing lung compliance (Matthay et al., 2019). Both atelectasis and reduced compliance cause an increase in the elastance and an alteration in the resistance of the pulmonary system. The changes in the elastance and resistance of the injured groups were the result of changes in the lung rather than the chest wall (Figure 3). We observed therapeutic hypothermia to be effective in reducing alveolar infiltrates which may have minimized pulmonary mechanical changes associated with lung injury. Diminishing the magnitude of mechanical alterations will likely attenuate additional stress on the lung tissue which may in turn prevent and protect against further lung injury and inflammation. Our findings are supported by previous research suggesting therapeutic hypothermia is effective in attenuating pulmonary mechanical changes in situations of VALI (Dostál et al., 2010).

Relative to the C group, animals in the I group experienced a decrease in EELV due to the aforementioned alterations in lung elastance and compliance (Figure 4b **)**. With a reduction in EELV, lung de‐recruitment increases and the alveoli become susceptible to collapse, which may facilitate further lung injury (Matthay & Zemans, 2011). ARDS can be exacerbated with the continuous distention and collapse of the alveoli which may happen with lower EELVs (Sahetya & Brower, 2017; Villar & Slutsky, 1993). A reduction in EELV may also cause greater work of breathing as the need for greater force generation and inspiratory muscle work during ventilation is required and may potentially enhance ARDS and lung injury (Dominelli & Sheel, 2012). With an increase in inflammation and injury, lung recruitment decreases causing a subsequent reduction in lung volume (Sahetya & Brower, 2017). We found little to no change in EELV in the HI group implying that hypothermia mitigated pulmonary mechanical alterations and diminished lung injury and severity.

With respect to blood gas findings, we found minimal differences between the I and HI groups. Therapeutic hypothermia induced a greater reduction in PaCO_2_ in the HI group compared to I group; however, we observed negligible differences in P/F ratio, pH and SaO_2_. Therapeutic hypothermia reduced PaCO_2_ by inducing little to no change in EELV and therefore the HI group was able to maintain adequate gas exchange. Additionally, we observed less injury and inflammation in the HI relative to I group which may explain the difference in PaCO_2_ and no difference in pH observed between the injured groups. No difference in P/F ratio and SaO_2_ may be due to the ability of hypothermia to improve oxygen supply and demand thereby maintaining PaO_2_ and SaO_2_ at appropriate levels (Sinclair & Andrews, 2010). Of note, SaO_2_ was lower in the HI group relative to I group at 12 hours following baseline which is contradictory to the ability of hypothermia to left‐shift the oxyhemoglobin dissociation curve; however, two animals in the HI group had very low SaO_2_ levels at this time point (~80–85%) with the rest of the animals completely saturated (95–100%). Moreover, cardiac output was not different among groups which is supported by recent work suggesting that cardiac output is not impacted by experimental ARDS (Henderson et al., 2014).

Additionally, we observed clear differences between the C and HC groups regarding our blood gas findings. We found that therapeutic hypothermia increased the P/F ratio in the HC group compared to C group. A higher P/F ratio was expected as therapeutic hypothermia can slow metabolism and improve oxygen supply and demand ratios, thereby left shifting the oxyhemoglobin disassociation curve (Coetzee & Swanepoel, 1990). Therapeutic hypothermia did not induce changes in PaCO_2_, pH or SaO_2_ in the C and HC groups.

### 4.5 Technical considerations

Our experimental model has several technical considerations that merit discussion. First, our study was relatively short in duration as the animals were euthanized at 10 h following the induction of injury. This short duration is a limitation to our study's generalizability as a significant portion of the development and progression of human ARDS occurs between 24 and 72 h following injury (Matthay et al., 2019). Since the animals were euthanized shortly after injury induction, we were unable to investigate how therapeutic hypothermia mitigates later stage ARDS and if it is effective in reducing mortality. Nonetheless, we demonstrate an attenuation of histological injury in the HI group suggesting a reduction in ARDS progression; however, there was no improvement in gas exchange. Second, the hypothermic animals were not rewarmed to baseline core temperatures; therefore, we were unable to determine if the benefits of therapeutic hypothermia remain after rewarming. Third, the hypothermic animals were chilled at the time of injury induction yet target core temperature was not achieved until after injury occurred. Inducing hypothermia and injury at the same time is unlikely to occur in a clinical setting; however, the animals in the HI group were injured for ~45 min before target core temperature was achieved (32°C). Fourth, the degree of injury induction was minor compared to human ARDS; however, the degree of injury achieved was sufficient to demonstrate the effectiveness of therapeutic hypothermia. Fifth, our metabolomic analysis distinguished clear differences between all four groups. Whether the metabolites and intermediates analyzed in the present study cause or are the result of the development of inflammation due to ARDS cannot be determined based on the scope of this study. Nevertheless, the present findings suggest clear differences in the metabolite profiles expressed in the four groups strongly suggesting these changes in the metabolic and inflammatory processes are caused by ARDS. Sixth, all animals underwent four BAL fluid collections prior to histological sampling; therefore, the histological findings may not be a true representation of lung status as this procedure can cause minor alterations in lung homeostasis. Lastly, ^1^H‐NMR spectroscopy is not as sensitive as mass spectroscopy and is limited to measuring metabolites at the micromolar concentration rather than nanomolar or picomolar concentrations see in gas chromatography–mass spectrometry (GC–MS) or liquid chromatography–mass spectrometry (LC–MS), respectively. Additionally, ^1^H‐NMR examines very few lipid metabolites which may be involved in the injury or protective responses. A strength of our experimental design is the use of conservative mechanical ventilation. The animals received limited tidal volumes of approximately 4–6 cc kg^−1^, which does not treat ARDS itself, rather it decreases the rate of new injury caused by mechanical ventilation (Acute Respiratory Distress Syndrome Network, 2000). The use of limited tidal volumes ensures that the injury induced is primarily from the oleic acid injection rather than from VALI. Much of the animal literature regarding ARDS has not controlled for the effects of injurious mechanical ventilation and VALI (Ganter and Hensel, 1997). This is of key importance as inappropriate tidal volumes during mechanical ventilation for ARDS have been shown to increase the expression of pro‐inflammatory genes (Wang et al., 2012).

## 5 CONCLUSION

We demonstrated that therapeutic hypothermia can reduce some markers of injury and inflammation associated with ARDS. Specifically, therapeutic hypothermia was effective in mitigating the histological changes associated with ARDS, was able to alter the metabolic profiles and attenuate the production of pro‐inflammatory cells responsible for ARDS and protected against pulmonary mechanical changes associated with ARDS. Taken together, our findings suggest that therapeutic hypothermia may attenuate acute injury progression in ARDS. Our findings provide the experimental basis for further studies designed to determine the effect of therapeutic hypothermia on longer term outcomes of ARDS and mortality prior to its use in clinical settings.

## AUTHOR CONTRIBUTIONS

Conceived and designed research: WRH, DEGG, MP, AWS, BWW, and PBD; Performed experiments: WRH, MMB, YM, CMP, HRP, DEGG, MS, AWS, BWW, and PBD; Analyzed data: SAA, WRH, MMB, YM, CMP, HRP, BWW, and PBD; Interpreted results and prepared figures: SAA, WRH, MMB, BWW, and PBC. Drafting and approval of final manuscript: All authors.

## Supporting information



Supplementary Material
