# Supplementary material for: Therapeutic hypothermia attenuates physiologic, histologic, and metabolomic markers of injury in a porcine model of acute respiratory distress syndrome
Source: Physiol Rep. 2022 May 4;10(9):e15286. doi: 10.14814/phy2.15286 (PMC9069168; doi:10.14814/phy2.15286)
Supplement: Supplementary file 1 — Supplementary Material [file PHY2-10-e15286-s001.pdf]

Therapeutic Hypothermia Attenuates Physiologic, Histologic and Metabolomic Markers of Injury in a Porcine Model of Acute Respiratory Distress Syndrome

*****Sarah A. Angus[1], *****William R. Henderson[2], Mohammad M. Banoei[3], Yannick Molgat-Seon[4], Carli M. Peters[5], Hanna R. Parmar[5], Donald E. G. Griesdale[2, 6], Mypinder Sekhon[2], A. William Sheel[5], Brent W. Winston[7, 8], Paolo B. Dominelli[1]

***, Contributed equally**

Affiliations

[1] Department of Kinesiology, University of Waterloo, Waterloo, Ontario, Canada
[2] Division of Critical Care Medicine, Department of Medicine, Faculty of Medicine, University of British Columbia, Vancouver, British Columbia, Canada
[3] Department of Critical Care Medicine, University of Calgary, Calgary, Alberta, Canada
[4] Department Kinesiology and Applied Health, University of Winnipeg, Winnipeg, Manitoba, Canada
[5] School of Kinesiology, University of British Columbia, Vancouver, British Columbia, Canada
[6] Department of Anesthesiology, Pharmacology & Therapeutics, University of British Columbia, Vancouver, British Columbia, Canada
[7] Department of Critical Care Medicine, University of Calgary, Calgary, Alberta, Canada
[8] Departments of Medicine and Biochemistry & Molecular Biology, University of Calgary, Calgary, Alberta, Canada

Correspondence

Paolo Dominelli, PhD
Department of Kinesiology, University of Waterloo
200 University Ave
Waterloo, Ontario, Canada
paolo.dominelli@uwaterloo.ca

Author Contributions:

Conceived and designed research: WRH, DEGG, MP, AWS, BWW, PBD; Performed experiments: WRH, MMB, YM, CMP, HRP, DEGG, MS, AWS, BWW, PBD; Analyzed data: SAA, WRH, MMB, YM, CMP, HRP, BWW, PBD; Interpreted results and prepared figures: SAA, WRH, MMB, BWW, PBC. Drafting and approval of final manuscript: All authors.

Funding:

The study was funded by a grant from Vancouver Coastal Health Research Institute (WRH). BWW was funded by a Grant from the Lung Association of Alberta of Alberta and the NWT and the Canadian Intensive Care Foundation (CICF). DEGG is funded through a Health Professional Investigator Award from the Michael Smith Foundation for Health Research.

Running Head: Therapeutic Hypothermia in Acute Respiratory Distress Syndrome

Group	Animal ID	Histological Injury Score Variables				
		**A**	**B**	**C**	**D**	**E**
Normothermic Control (C)	101	5	20	0	0	8
	102	0	20	0	0	3
	103	4	30	0	0	26
	104	0	23	0	0	10
	105	0	20	0	0	13
Normothermic Injured (I)	201	39	40	4	6	0
	202	40	40	21	23	7
	203	40	40	17	19	9
	204	38	39	15	15	8
	205	33	36	4	30	11
Hypothermic Injured (HI)	301	33	39	2	15	2
	302	19	26	0	14	0
	303	10	24	0	2	0
	304	27	32	0	4	4
	305	34	40	0	9	23
Hypothermic Control (HC)	401	0	39	0	0	22
	402	0	22	0	0	0
	403	0	35	0	0	20
	404	0	40	0	0	17
	405	5	39	0	0	19

Table S1. Histological lung injury score variables for each animal in every group. **A:** Neutrophils in the alveolar space. **B:** Neutrophils in the interstitial space. **C:** Hyaline membranes. **D:** Proteinaceous debris filling the airspaces. **E:** Alveolar septal thickening.

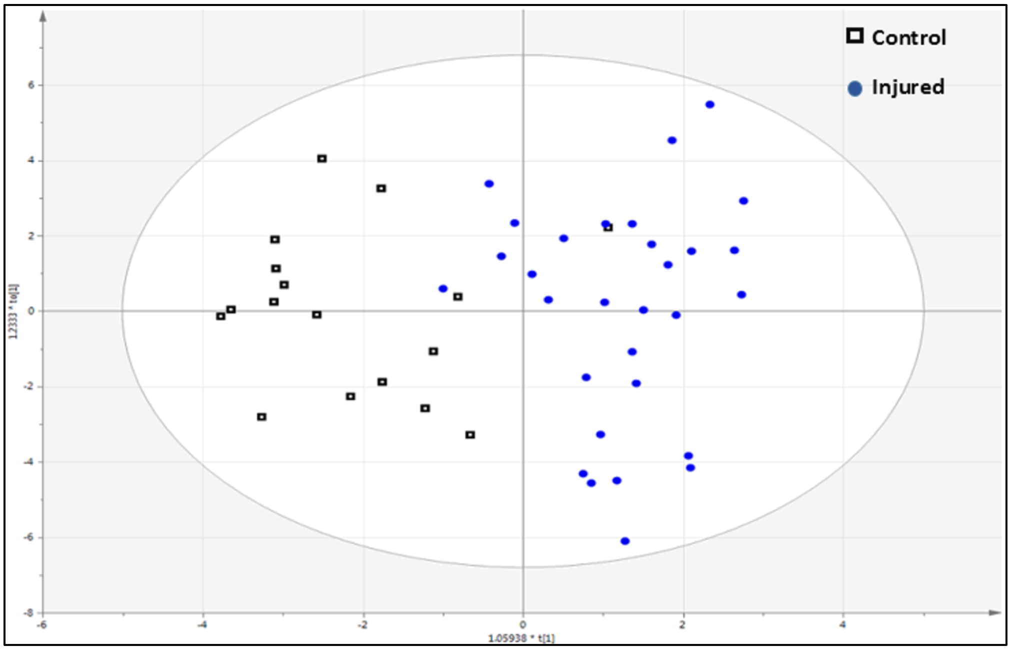

Figure S1. OPLS-DA discrimination model shows a very good separation between I and C cohorts. The Q^2= 0.4 and p=0.00029 demonstrate the predictable and significant separation model based on 26 most differentiating metabolites.

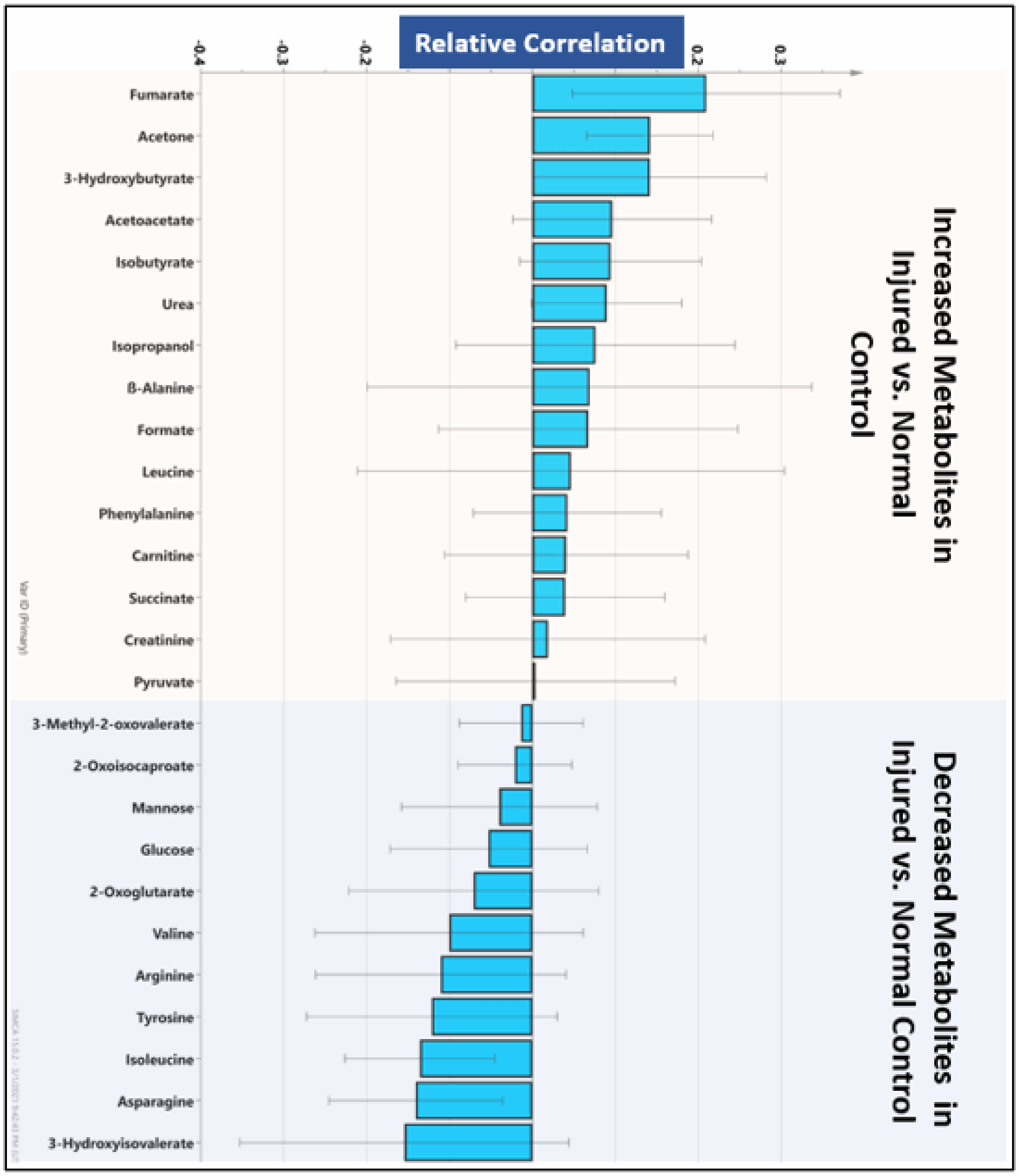

Figure S2. Coefficient plot illustrates the relative correlation of increased and decreased most differentiating metabolites between I and C cohorts.

Name	Mean (SD) of Control	Mean (SD) of Injured	p-value	Fold Change	Control vs. Injured.
Isoleucine	0.108 (0.029)	0.080 (0.022)	0.0005	1.35	Down
Pyruvate	0.039 (0.013)	0.029 (0.009)	0.0038	1.35	Down
Valine	0.024 (0.005)	0.021 (0.004)	0.0051	1.18	Down
Tyrosine	0.082 (0.022)	0.067 (0.017)	0.0105	1.23	Down
Asparagine	0.027 (0.008)	0.022 (0.003)	0.0165	1.23	Down
Methionine	0.033 (0.009)	0.028 (0.006)	0.0324	1.2	Down
Leucine	0.016 (0.004)	0.013 (0.003)	0.038	1.2	Down
Lysine	0.015 (0.003)	0.013 (0.003)	0.0466	1.15	Down
3-Hydroxyisovalerate	0.001 (0.000)	0.001 (0.000)	0.0005 (W)	1.45	Down
3-Methyl-2-oxovalerate	0.015 (0.004)	0.011 (0.005)	0.0158 (W)	1.3	Down
3-Hydroxybutyrate	0.038 (0.012)	0.059 (0.031)	0.0028	-1.53	Up
Succinate	0.004 (0.001)	0.005 (0.002)	0.0201	-1.25	Up
Acetone	0.005 (0.002)	0.006 (0.003)	0.0384 (W)	-1.44	Up

Table S2. T-test analysis shows the metabolites significantly changed between I and C cohorts.

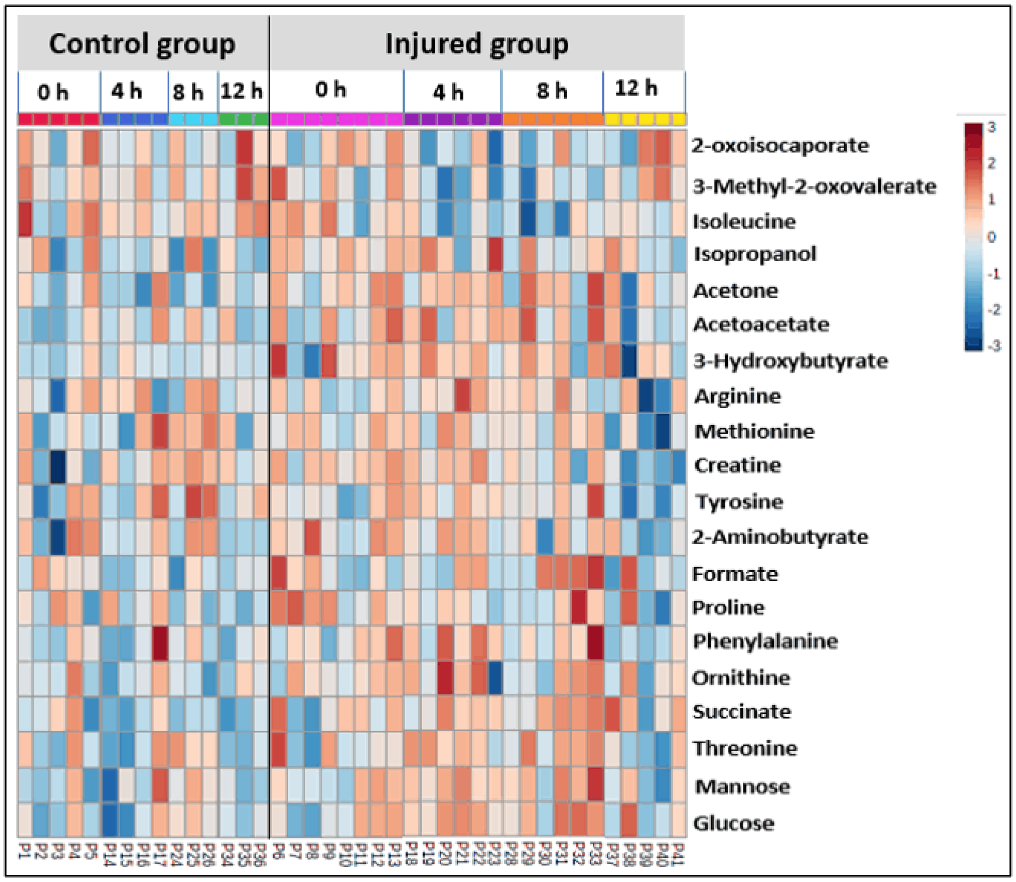

Figure S3. Heatmap analysis reveals the overtime metabolites changes of I and C cohort. Metabolite changes are remarkably higher in I cohort compared to C cohort with highest points at 4 and 8 hours after injury.

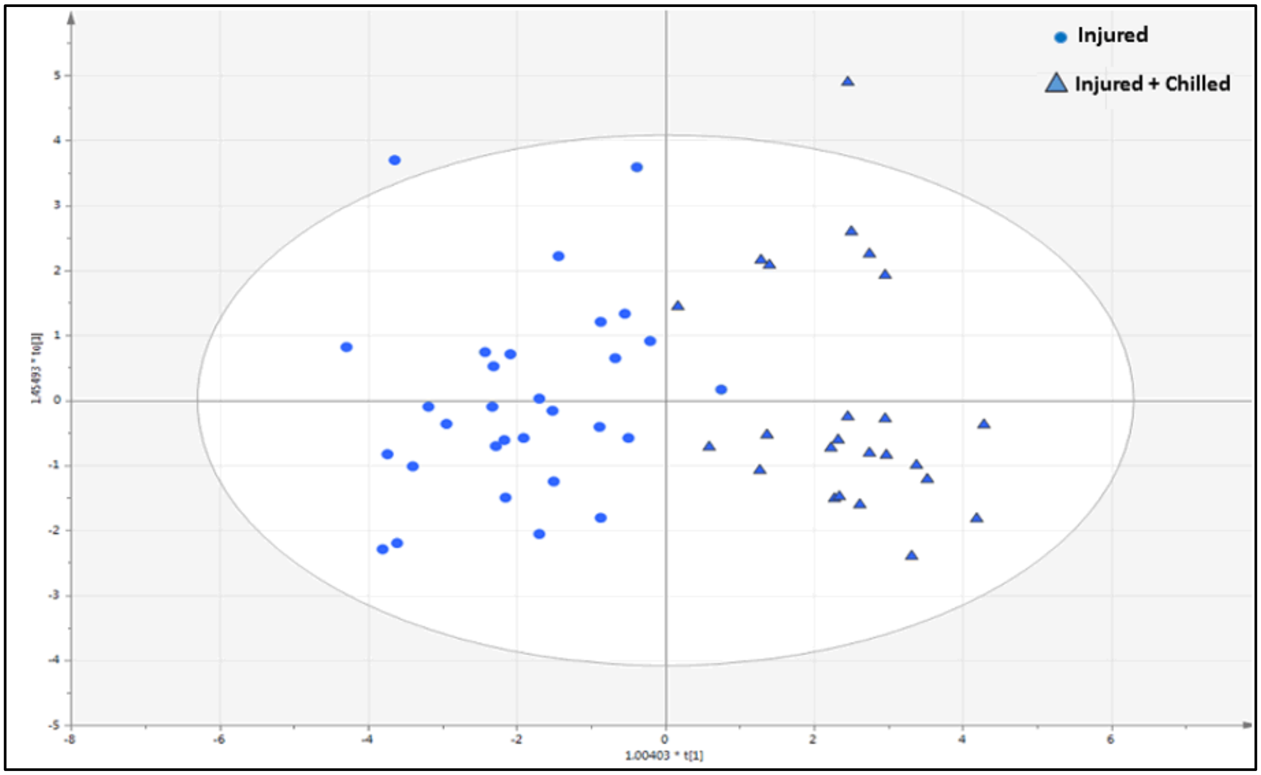

Figure S4. OPLS-DA discrimination model shows a very good separation between HI and I cohorts. The Q2= 0.64 and $p=2.2\times10^{-10}$ demonstrate highly predictable and significant separation model based on the 20 most differentiating metabolites.

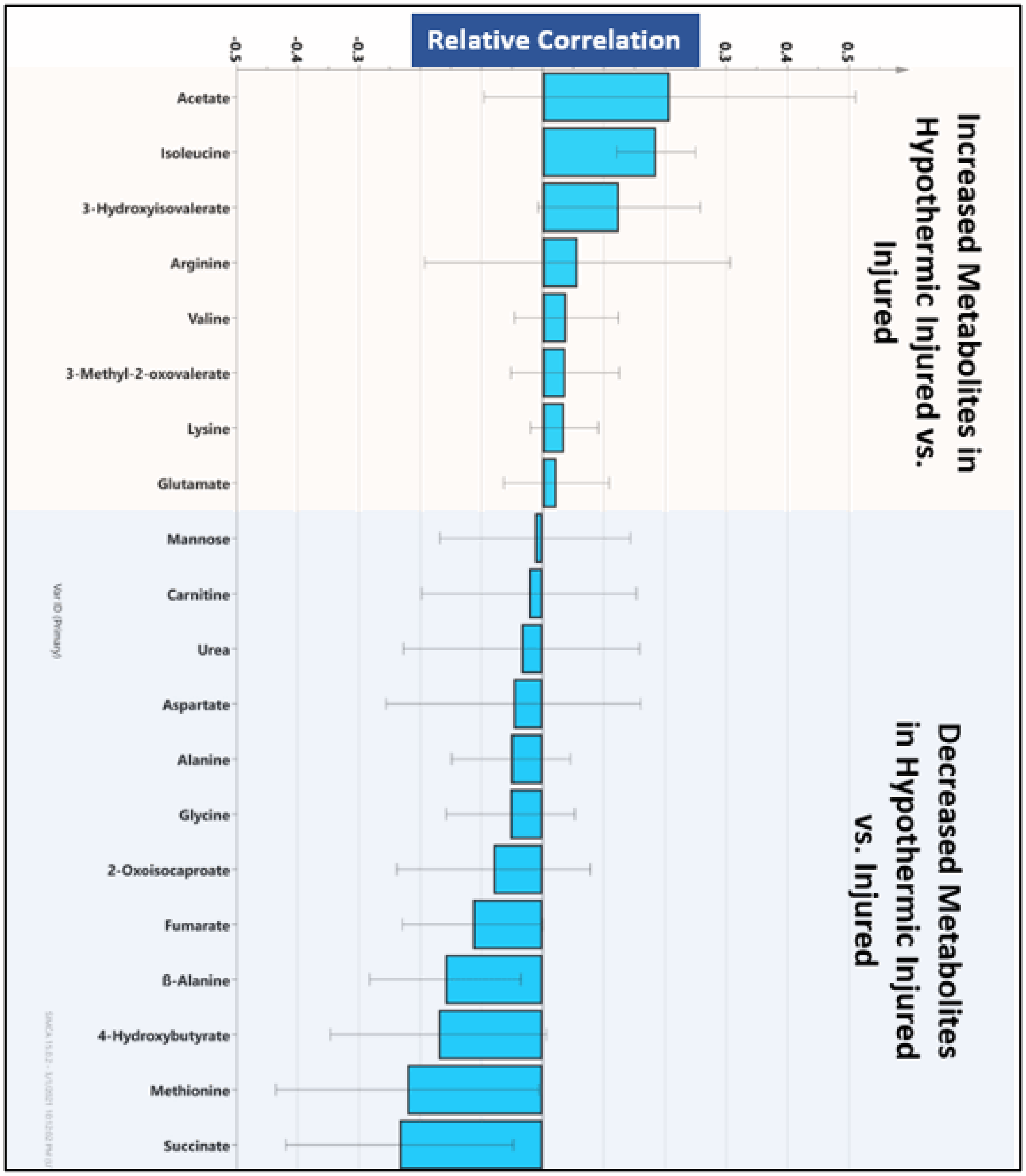

Figure S5. Coefficient plot illustrates the relative correlation of increased and decreased most differentiating metabolites between HI and I cohorts.

	Name	Mean (SD) of I cohort	Mean (SD) of HI cohort	p-value	Fold Change	HI cohort vs. I cohort
1	2-Aminobutyrate	0.075 (0.017)	0.104 (0.030)	0.0002	-1.39	Up
2	Arginine	0.122 (0.031)	0.168 (0.046)	0.0002	-1.38	Up
3	Valine	0.021 (0.004)	0.026 (0.006)	0.0003	-1.27	Up
4	Leucine	0.013 (0.003)	0.017 (0.005)	0.0008	-1.3	Up
5	Tyrosine	0.067 (0.017)	0.084 (0.022)	0.0027	-1.25	Up
6	2-Hydroxyisovalerate	0.004 (0.002)	0.005 (0.002)	0.0381	-1.25	Up
7	2-Hydroxybutyrate	0.047 (0.022)	0.060 (0.024)	0.044	-1.27	Up
8	3-Methyl-2-oxovalerate	0.011 (0.005)	0.016 (0.005)	0.0011 (W)	-1.42	Up
9	3-Hydroxyisovalerate	0.001 (0.000)	0.002 (0.001)	0.0013 (W)	-1.49	Up
10	Acetate	0.058 (0.021)	0.226 (0.612)	0.0023 (W)	-3.87	Up
11	2-Oxoisocaproate	0.013 (0.005)	0.017 (0.006)	0.0079 (W)	-1.37	Up
12	Serine	0.010 (0.003)	0.011 (0.002)	0.0482 (W)	-1.08	Up
13	Alanine	0.019 (0.004)	0.016 (0.003)	0.0031	1.19	Down
14	Glutamate	0.090 (0.024)	0.072 (0.019)	0.0046	1.25	Down
15	b-Alanine	0.004 (0.001)	0.003 (0.001)	0.0257	1.27	Down
16	Glycine	0.009 (0.002)	0.006 (0.001)	< 0.0001 (W)	1.39	Down
17	Urea	0.048 (0.045)	0.026 (0.021)	0.0051 (W)	1.83	Down
18	Succinate	0.005 (0.002)	0.005 (0.002)	0.0127 (W)	1.2	Down
19	Carnitine	0.005 (0.002)	0.004 (0.002)	0.0205 (W)	1.26	Down
20	4-Hydroxybutyrate	0.010 (0.012)	0.005 (0.002)	0.0480 (W)	2.04	Down

Table S3. T-test analysis shows the metabolites significantly changed between HI and I cohorts

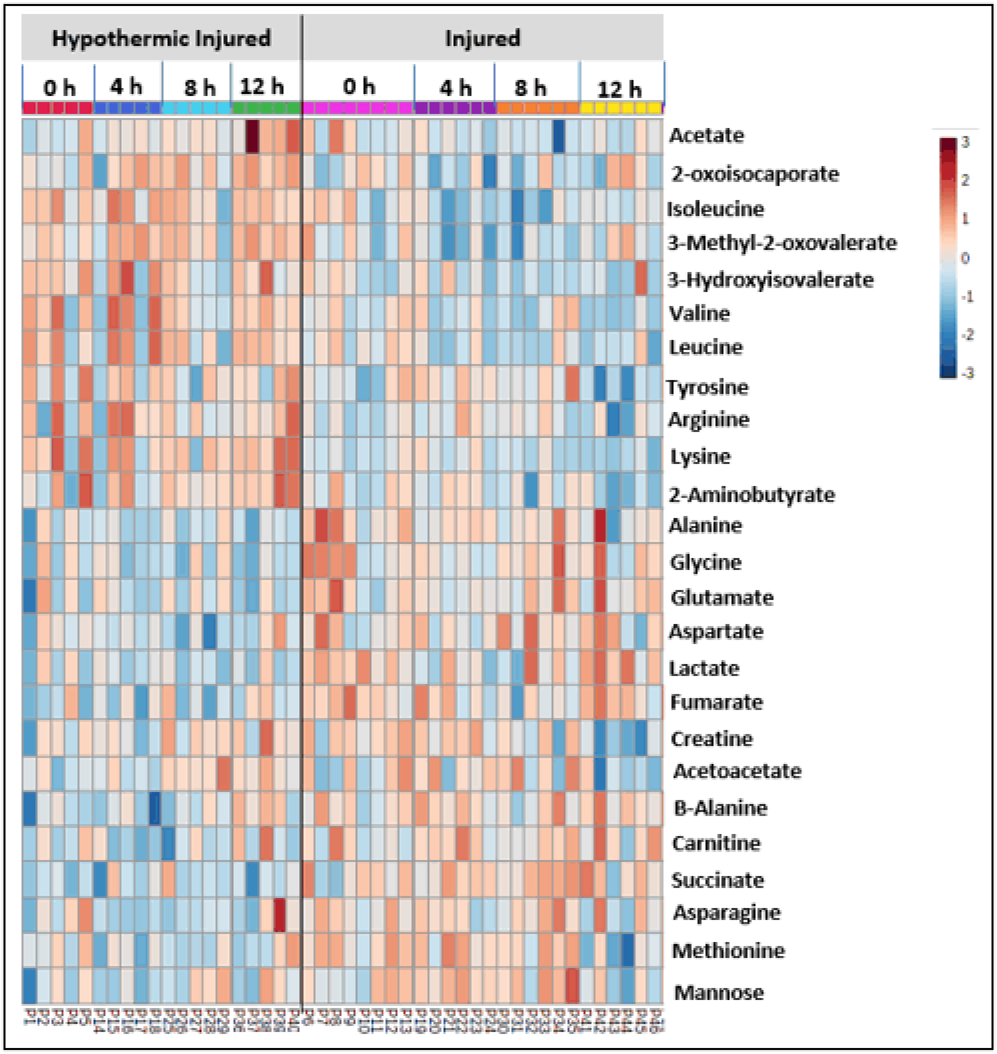

Figure S6. Heatmap analysis reveals the overtime metabolites changes of I and C cohort. Metabolite changes are remarkably higher in HI cohort compared to I cohort.

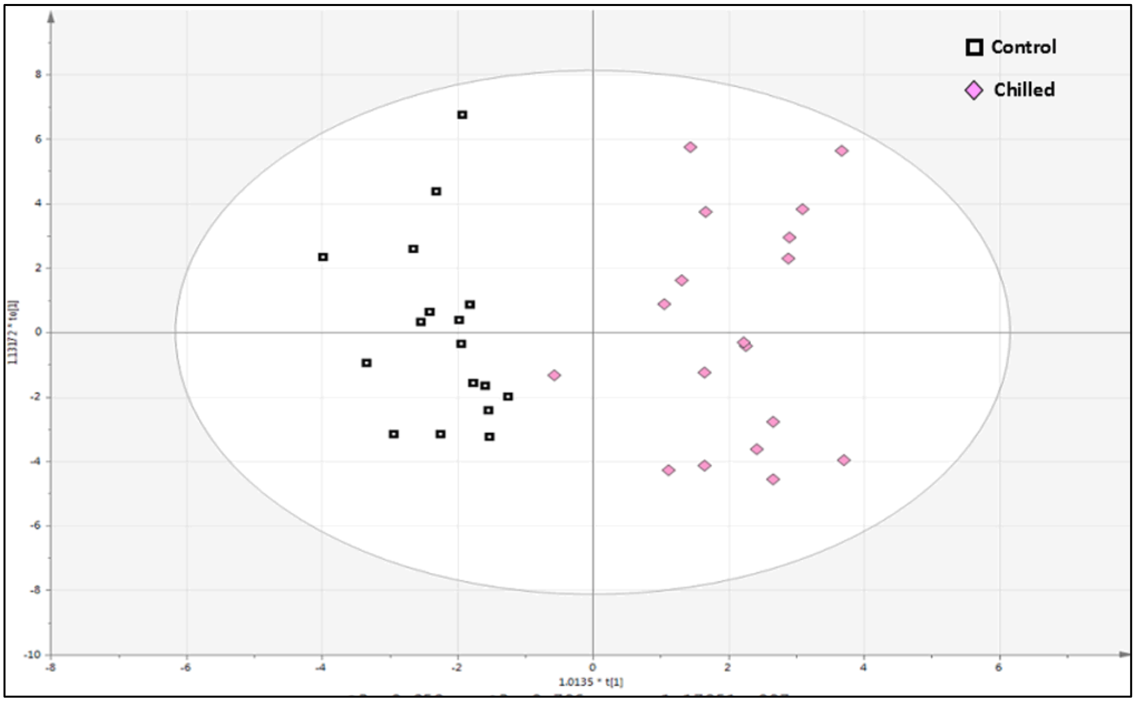

Figure S7. OPLS-DA discrimination model shows a very good separation between H and C cohorts. The Q2= 0.8 and $p=2.2\times10^{-10}$ demonstrate highly predictable and significant separation model based on the 26 most differentiating metabolites.

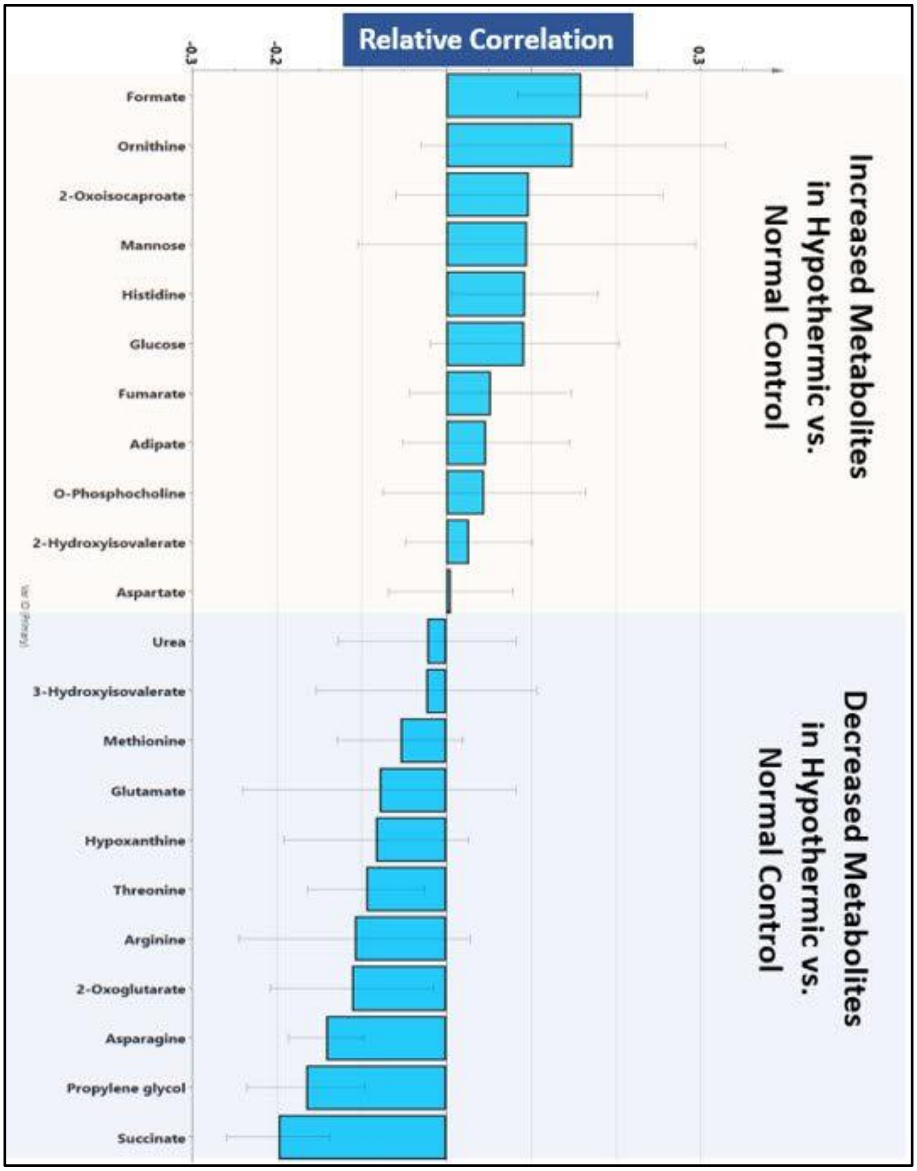

Figure S8. Coefficient plot illustrates the relative correlation of increased and decreased most differentiating metabolites between H and C cohorts.

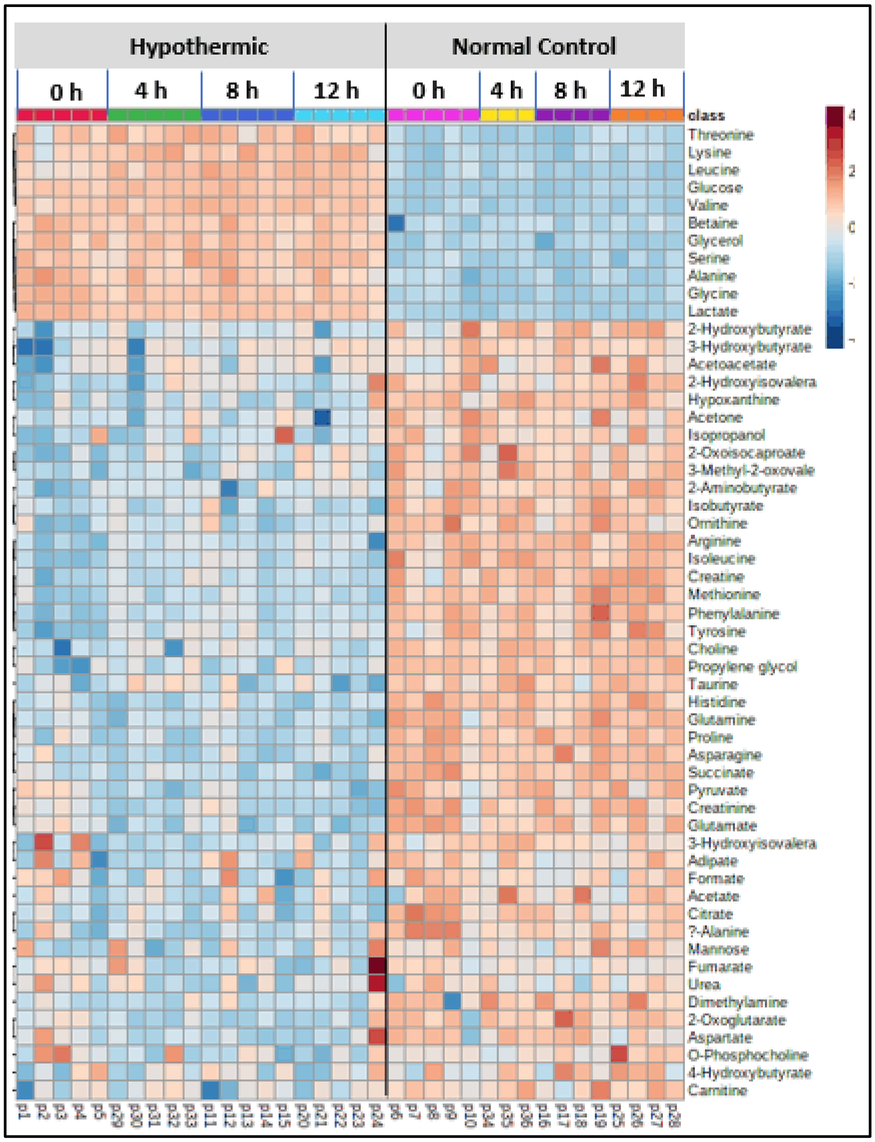

Figure S9. Heatmap analysis reveals the overtime metabolites changes of I and C cohort. Metabolite changes are remarkably higher in H cohort compared to C cohort.

	Name	Mean (SD) of Chilled	Mean (SD) of Control	p-value	Fold Change	Chilled/Control
1	Arginine	0.094 (0.033)	0.139 (0.030)	0.0001	-1.48	Down
2	Proline	0.070 (0.016)	0.090 (0.013)	0.0002	-1.29	Down
3	Methionine	0.024 (0.006)	0.033 (0.009)	0.0007	-1.38	Down
4	Glutamate	0.067 (0.019)	0.090 (0.021)	0.0014	-1.34	Down
5	Glutamine	0.129 (0.022)	0.156 (0.032)	0.0052	-1.21	Down
6	Creatine	0.199 (0.039)	0.245 (0.061)	0.009	-1.23	Down
7	Choline	0.006 (0.002)	0.008 (0.002)	0.0199	-1.24	Down
8	Isoleucine	0.088 (0.022)	0.108 (0.029)	0.0205	-1.23	Down
9	2-Hydroxybutyrate	0.038 (0.015)	0.054 (0.025)	0.0269	-1.42	Down
10	Alanine	0.174 (0.052)	0.021 (0.005)	< 0.0001	8.42	Up
11	Betaine	0.139 (0.030)	0.020 (0.008)	< 0.0001	6.93	Up
12	Glucose	2.478 (0.563)	0.049 (0.013)	< 0.0001	50.07	Up
13	Glycerol	0.669 (0.271)	0.013 (0.006)	< 0.0001	50.02	Up
14	Glycine	0.356 (0.120)	0.009 (0.003)	< 0.0001	40.35	Up
15	Lactate	0.605 (0.157)	0.016 (0.005)	< 0.0001	38.42	Up
16	Leucine	0.126 (0.038)	0.016 (0.004)	< 0.0001	8.01	Up
17	Lysine	0.135 (0.058)	0.015 (0.003)	< 0.0001	9.02	Up
18	Propylene glycol	0.012 (0.009)	0.032 (0.006)	< 0.0001	-2.7	Down
19	Serine	0.093 (0.020)	0.011 (0.002)	< 0.0001	8.61	Up
20	Succinate	0.002 (0.001)	0.004 (0.001)	< 0.0001	-2.02	Down
21	Threonine	0.094 (0.028)	0.014 (0.003)	< 0.0001	6.98	Up
22	Valine	0.208 (0.041)	0.024 (0.005)	< 0.0001	8.52	Up

23	Asparagine	0.020 (0.007)	0.027 (0.008)	0.0011 (W)	-1.37	Down
24	Formate	0.016 (0.006)	0.011 (0.002)	0.0012 (W)	1.47	Up
25	Isobutyrate	0.005 (0.002)	0.007 (0.002)	0.0124 (W)	-1.29	Down
26	Dimethylamine	0.001 (0.000)	0.002 (0.001)	0.0126 (W)	-1.27	Down
27	Citrate	0.029 (0.013)	0.041 (0.016)	0.0223 (W)	-1.41	Down
28	?-Alanine	0.003 (0.002)	0.004 (0.001)	0.0259 (W)	-1.24	Down
29	2-Oxoglutarate	0.015 (0.008)	0.019 (0.010)	0.0354 (W)	-1.29	Down
30	Hypoxanthine	0.011 (0.006)	0.013 (0.003)	0.0354 (W)	-1.17	Down
31	Isopropanol	0.013 (0.018)	0.013 (0.008)	0.0411 (W)	-1.05	Down
32	Acetone	0.003 (0.002)	0.005 (0.002)	0.0425 (W)	-1.49	Down

Table S4. T-test analysis shows the metabolites significantly changed between H and C cohorts

Injured Group vs. Control Group		Chilled Group vs. Control Group	
Increased	Decreased	Increased	Decreased
Fumarate	Glucose	Threonine	Histidine
Urea	Arginine	Betaine	Ornithine
Leucine	Asparagine	Arginine	Adipate
Phenylalanine		Citrate	O-phosphocholine
Creatinine		Hypoxanthine	Pyruvate
Pyruvate			Formate

Table S5. Most differentiating metabolites that specifically changed due to Injury and hypothermic intervention

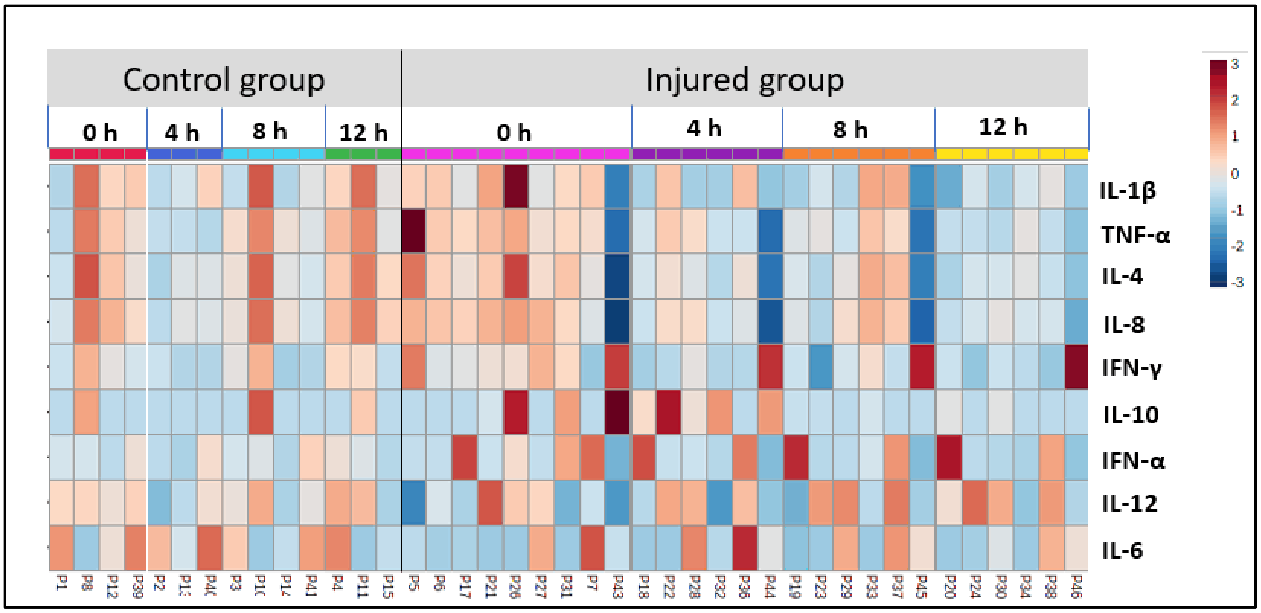

Figure S10. Heatmap analysis reveals the overtime metabolites changes of I and C cohort. Metabolite changes are remarkably higher in I cohort compared to C cohort.

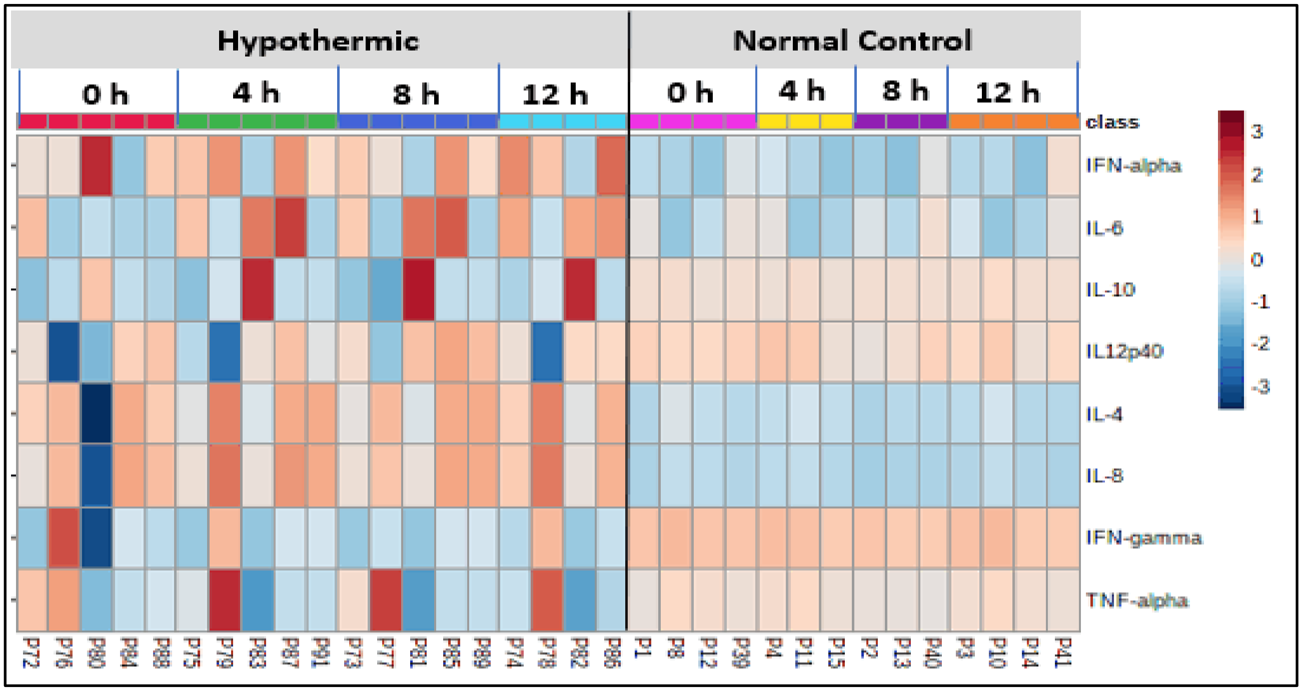

Figure S11. Heatmap analysis reveals the overtime metabolites changes of I and C cohort. Metabolite changes are remarkably higher in H cohort compared to C cohort.
